# P-1282. Saving Lives and Costs: Respiratory Syncytial Virus Wastewater Surveillance to Guide all infant prophylaxis in Ontario, Canada

**DOI:** 10.1093/ofid/ofae631.1463

**Published:** 2025-01-29

**Authors:** Elisabeth Mercier, Nisha Thampi, Shen Wan, Bosco Paes, John Fullarton, Ian Keary, Barry Rodgers-Gray, Robert Delatolla

**Affiliations:** Univeristy of Ottawa, Ottawa, Ontario, Canada; CHEO, Ottawa, Ontario, Canada; University of Ottawa, Ottawa, Ontario, Canada; McMaster University - Department of Pediatrics (Neonatal Division), Hamilton, Ontario, Canada; Violicom Medical Limited, Aldermaston, England, United Kingdom; Violicom Medical Limited, Aldermaston, England, United Kingdom; Violicom Medical Limited, Aldermaston, England, United Kingdom; Univeristy of Ottawa, Ottawa, Ontario, Canada

## Abstract

**Background:**

Wastewater-based surveillance (WBS) of respiratory syncytial virus (RSV) detected seasonal activity up to one month earlier than clinical surveillance (CS) during the 2022 RSV season in two Ontario cities, Canada. The associated cost-consequence analysis comparing the use of WBS vs CS to guide the start of provincial immunoprophylaxis programs showed WBS-guided prophylaxis realized savings of CAD $2.1-3.5m in the first year and $13.7-16.6m over 1-3 years due to early RSV detection. Further investigation is needed to explore the reproducibility and scalability of WBS for RSV in a provincial surveillance initiative.

Geospatial distribution of the 12 sentinel sampling locations across Ontario, Canada

**Figure:**
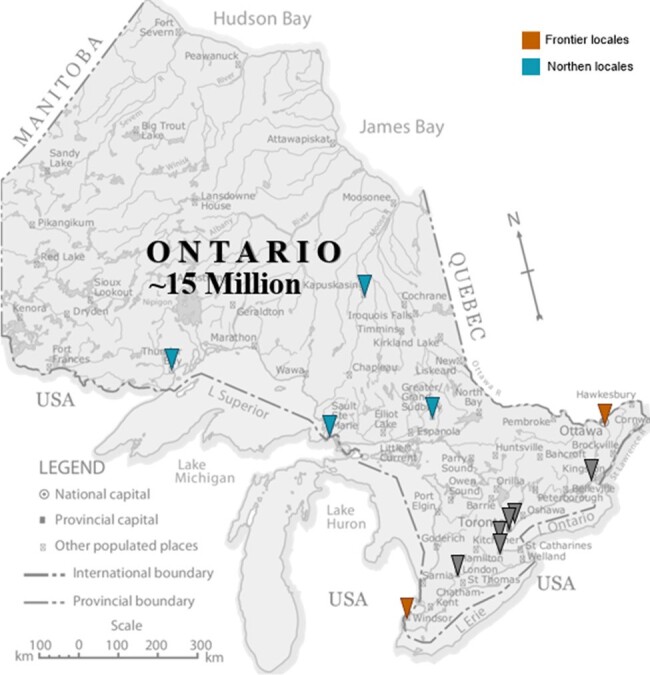

**Methods:**

In collaboration with the Ontario Ministry of Health (MOH), 12 locations in the province were selected as sentinel WBS sites based on population density, socio-economic strata, and their significance as primary pediatric centres where palivizumab is administered (Figure 1). City-wide, 24-hour composite wastewater samples were collected and screened for RSV by RT-qPCR three to seven times a week. We monitored RSV-WBS data and pediatric RSV-related hospitalizations (RSVH) between September 1, 2023, to March 15, 2024.

Cumulative fraction of locations for the onset of the 2023 RSV season in Ontario, Canada

**Figure:**
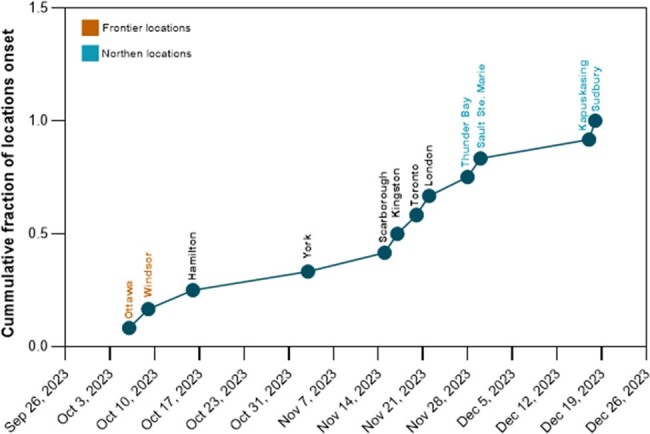

Date of onset of regional RSV activity as identify by wastewater surveillance at each of the 12 sentinel locations during the 2023 RSV season, represented as a cumulative fraction.

**Results:**

During the study period, RSV was detected in 76% (1092/1440) of all wastewater samples collected, with detection at all locations. WBS effectively tracked the geographical variation in viral activity across the province. There was a 73-day variation in the RSV season onset across locations, with northern sites lagging southern locations by 62 ± 11 days (Figure 2). WBS detected regional RSV activity up to 15 days prior to the provincial start date of October 30, 2023, as determined by CS. Considering the estimated WBS cost of $0.50 per eligible infant compared to an average of $5,000 per RSVH, and an estimated fewer RSVHs per year, provincial WBS-guided prophylaxis could significantly reduce healthcare costs and the burden of RSV associated illness.

**Conclusion:**

RSV WBS could be a highly cost-effective strategy that identifies the early onset of the RSV season, thereby optimizing immunoprophylaxis timing. Additionally, it is scalable to communities of varying sizes and socioeconomic strata and reflects geospatial variation in regional RSV activity supporting its use in a provincial-wide WBS initiative.

**Disclosures:**

**Bosco Paes, MD, Professor Emeritus**, AstraZeneca: advisor/lecturer outside the scope of this study|Sanofi: advisor/lecturer outside the scope of this study **John Fullarton, n/a**, AstraZeneca: Employer has received payment from AstraZeneca for work on various projects outside the scope of this study|Sanofi: Employer has received payment from Sanofi for work on various projects outside the scope of this study **Barry Rodgers-Gray, n/a**, AstraZeneca: Employer has received payment from AstraZeneca for work on various projects outside the scope of this study|Sanofi: Employer has received payment from Sanofi for work on various projects outside the scope of this study

